# Supplementary material for: Dynamic transcriptomic profiles of zebrafish gills in response to zinc supplementation
Source: BMC Genomics. 2010 Oct 11;11:553. doi: 10.1186/1471-2164-11-553 (PMC3091702; doi:10.1186/1471-2164-11-553)
Supplement: Additional file 2 — Interactive Direct Interaction Network representing the molecular interactions between zinc, copper, iron, calcium and proteins encoded by transcripts changed by zinc supplementation. Mini web-site containing index.html and hyperlinked pages in subdirectory describing a Direct Interaction Network automatically generated based on curated interactions contained within the proprietary PathwayArchitect database. Ovals represent proteins and the circles symbolize metal ions. Objects are coloured by their abundance in zebrafish at the time-point they were significantly different from the control is a scale from -4 fold (dark green) to +4 fold (dark red). Where significant differences were found at more than one time-point, the colour overlay shows expression at the first instance. Dark blue squares denote 'binding', and light blue squares 'expression'; green squares stand for 'regulation', green diamonds for 'metabolism', and green circles for 'promoter binding'. Arrow heads indicate directionality of the interaction where annotated. All nodes and edges can be further interrogated by selecting the relative area of the image. [file 1471-2164-11-553-S2.zip › PathwayArchitect Zn xs DIN/957047.html]

# REGULATION:

|  |  |
| --- | --- |
| Type | REGULATION |
| Effect | None |


---

|  |  |
| --- | --- |
| Score | 0 |


---

|  |  |
| --- | --- |
| Reference Count | 5 |


---

|  |  |
| --- | --- |
| Mechanism | Unknown |


---

|  |  |
| --- | --- |
| Reference:0 || Sentence | "A prospective study of 149 unselected incident cases of pancreatic adenocarcinoma and 146 ethnically-matched controls found no associations between GSTM1 (adjusted odds ratio (AOR) 1.14), GSTT1 (AOR: 1.19) and CYP1A1 (AOR: 1.08) polymorphisms and pancreatic cancer susceptibility." |
| PMID | 10817498 |
| Year | 2000 |
| Species | Human |
| Journal | Br J Cancer |
| RefScore | 1 |
| Source | PArchNLP |
  |
|  |  |


---

|  |  |
| --- | --- |
 Reference:1 || Sentence | "No associations between GSTM1, GSTM3, GSTT1 or CYP1A1 genotypes and susceptibility or, time to node metastases were identified." |
| PMID | 9825835 |
| Year | 1998 |
| Species | Human |
| Journal | Pharmacogenetics |
| RefScore | 1 |
| Source | PArchNLP |
  ||  |  |


---

|  |  |
| --- | --- |
 Reference:2 || Sentence | "Associations between genetic polymorphisms of CYP1A1, CYP1A2, CYP2E1, GSTM1 and GSTT1 and prostate cancer (PCa) were analyzed in a case-control study of 315 individuals." |
| PMID | 11275366 |
| Year | 2001 |
| Species | Human |
| Journal | Cancer Lett |
| RefScore | 0 |
| Source | PArchNLP |
  ||  |  |


---

|  |  |
| --- | --- |
 Reference:3 || Sentence | "In the present study, we examined possible associations between genetic polymorphisms of CYP1A1 MspI, GSTM1, and GSTT1 and colon cancer in a United Kingdom population." |
| PMID | 12210502 |
| Year | 2002 |
| Species | Human |
| Journal | Teratog Carcinog Mutagen |
| RefScore | 0 |
| Source | PArchNLP |
  ||  |  |


---

|  |  |
| --- | --- |
 Reference:4 || Sentence | Logistic regression and a log-linear model were used to analyze the association between genetic variants such as CYP1A1\*2A, CYP1A1\*2B, CYP1A1\*4, GSTT1, GSTM1, and XRCC3 and SGA. |
| Year | 2006 |
| PMID | 16357593 |
| Species | Human |
| Journal | Epidemiology |
| RefScore | 1 |
| Source | PArchNLP |
  |


---

|  |  |
| --- | --- |
